# Comparative safety of anti-epileptic drugs among infants and children exposed *in utero* or during breastfeeding: protocol for a systematic review and network meta-analysis

**DOI:** 10.1186/2046-4053-3-68

**Published:** 2014-06-25

**Authors:** Andrea C Tricco, Elise Cogo, Veroniki A Angeliki, Charlene Soobiah, Brian Hutton, Brenda R Hemmelgarn, David Moher, Yaron Finkelstein, Sharon E Straus

**Affiliations:** 1 Li Ka Shing Knowledge Institute, St. Michael’s Hospital, 209 Victoria Street, East Building, Toronto, Ontario M5B 1 T8, Canada; 2 Institute of Health Policy Management and Evaluation, University of Toronto, Health Sciences Building, 155 College Street, Suite 425, Toronto, Ontario M5T 3 M6, Canada; 3 Clinical Epidemiology Program, Centre for Practice-Changing Research, Ottawa Hospital Research Institute, The Ottawa Hospital – General Campus and University of Ottawa, 501 Smyth Road, Box 711, Ottawa, Ontario K1H 8 L6, Canada; 4 Departments of Medicine and Community Health Sciences, University of Calgary, TRW Building, 3rd Floor, 3280 Hospital Drive NW, Calgary, Alberta T2N 4Z6, Canada; 5 The Hospital for Sick Children, 555 University Avenue, Toronto, Ontario M5G 1X8, Canada; 6 Department of Pediatrics, University of Toronto, 172 St. George Street, Toronto, Ontario M5R 0A3, Canada; 7 Department of Pharmacology and Toxicology, University of Toronto, Medical Sciences Building, 1 King’s College Circle, Room 4207, Toronto, Ontario M5S 1A8, Canada; 8 Department of Geriatric Medicine, University of Toronto, 172 St. George Street, Toronto, Ontario M5R 0A3, Canada

**Keywords:** Anti-epileptic drug, Breastfeeding, Comparative safety, Congenital malformation, Epilepsy, Fetus, Infant, Network meta-analysis, Pregnancy, Systematic review

## Abstract

**Background:**

Epilepsy affects about 1% of the general population. Anti-epileptic drugs (AEDs) prevent or terminate seizures in individuals with epilepsy. Pregnant women with epilepsy may continue taking AEDs. Many of these agents cross the placenta and increase the risk of major congenital malformations, early cognitive and developmental delays, and infant mortality. We aim to evaluate the comparative safety of AEDs approved for chronic use in Canada when administered to pregnant and breastfeeding women and the effects on their infants and children through a systematic review and network meta-analysis.

**Methods:**

Studies examining the effects of AEDs administered to pregnant and breastfeeding women regardless of indication (e.g., epilepsy, migraine, pain, psychiatric disorders) on their infants and children will be included. We will include randomized clinical trials (RCTs), quasi-RCTs, non-RCTs, controlled before-after, interrupted time series, cohort, registry, and case-control studies. The main literature search will be executed in MEDLINE, EMBASE, and the Cochrane Central Register of Controlled Trials. We will seek unpublished literature through searches of trial protocol registries and conference abstracts. The literature search results screening, data abstraction, and risk of bias appraisal will be performed by two individuals, independently. Conflicts will be resolved through discussion. The risk of bias of experimental and quasi-experimental studies will be appraised using the Cochrane Effective Practice and Organization of Care Risk-of-Bias tool, methodological quality of observational studies will be appraised using the Newcastle-Ottawa Scale, and quality of reporting of safety outcomes will be conducted using the McMaster Quality Assessment Scale of Harms (McHarm) tool. If feasible and appropriate, we will conduct random effects meta-analysis. Network meta-analysis will be considered for outcomes that fulfill network meta-analysis assumptions.

The primary outcome is major congenital malformations (overall and by specific types), while secondary outcomes include fetal loss/miscarriage, minor congenital malformations (overall and by specific types), cognitive development, psychomotor development, small for gestational age, preterm delivery, and neonatal seizures.

**Discussion:**

Our systematic review will address safety concerns regarding the use of AEDs during pregnancy and breastfeeding. Our results will be useful to healthcare providers, policy-makers, and women of childbearing age who are taking anti-epileptic medications.

**Systematic review registration:**

PROSPERO CRD42014008925.

## Background

Epilepsy is the most common chronic neurological condition, affecting 0.6 to 1% of the population [1,2]. Individuals with uncontrolled epilepsy experience recurrent seizures, which can have psychosocial and physical consequences, including a compromised life expectancy [3,4]. The goal of anti-epileptic treatment is to improve quality of life and health outcomes by reducing the frequency of seizures [4].

Anti-epileptic medications decrease seizures by reducing excitation and enhancing inhibition of neurons [5-7]. Many of these medications target different channels, including calcium, sodium, and glutamate, and are broadly classified as first generation agents (e.g., phenobarbitone, phenytoin, carbamazepine, sodium valproate, ethosuximide) and second generation agents (e.g., lamotrigine, levetiracetam, topiramate, gabapentin, vigabatrin, oxcarbazepine, clobazam, clonazepam, zonisamide, lacosamide, rufinamide, primidone) [8]. Due to the broad and varied mechanisms of action, the indications for some of these medications also include pain syndromes, psychiatric disorders, and migraine headaches [8].

Many clinical practice guidelines recommend that women of childbearing age continue to take their anti-epileptic medications; however, medications with lower risk of teratogenic events are advised [9,10] since anti-epileptic drugs (AEDs) cross the placenta or transfer through breast milk, posing risks to the fetus and infant [9,11,12].

Some AEDs have been associated with increased risk of harm to the fetus and infants. For example, exposure to valproate has led to increased risk of major congenital malformations [10], cognitive delay, and minor congenital abnormalities [13-16]. Phenobarbital has been associated with minor congenital abnormalities and developmental delay [17,18]. Carbamazepine and lamotrigine have been associated with minor congenital abnormalities [19-22]. However, other than studies of the use of valproate, many studies have produced inconsistent findings regarding harm to the fetus and infant with use of other agents [23]. As such, our objective is to evaluate the comparative safety of AEDs for infants and children who were exposed *in utero* or during breastfeeding through a systematic review and network meta-analysis.

## Methods/Design

### Protocol

A systematic review protocol was developed and registered with the PROSPERO database (CRD42014008925, available at: http://www.crd.york.ac.uk/PROSPERO/display_record.asp?ID=CRD42014008925). It was revised with feedback from the decision-makers who posed the query within Health Canada, healthcare practitioners, content experts, and research methodologists. The reporting of our systematic review protocol was guided by the Preferred Reporting Items for Systematic Reviews and Meta-analyses Protocols [24].

### Eligibility criteria

We will include studies examining the effects of AEDs on infants and children who were exposed *in utero* or during breastfeeding. We will include experimental studies (randomized clinical trials [RCTs], quasi-RCTs, non-RCTs), quasi-experimental studies (controlled before and after studies, interrupted time series), and observational studies (cohort, case-control, registry studies) of pregnant women at any stage of pregnancy and breastfeeding women and their infants/children. The rationale for including other study designs in addition to RCTs is that there are ethical issues in conducting RCTs of AEDs in pregnancy, so RCT evidence might not exist for some or all of these drugs. Given that our review includes rare outcomes, including observational evidence is crucial. In contrast to efficacy evaluation, safety assessment usually requires very large sample sizes to be able to detect adverse events. Therefore, while RCTs have lower risk of bias, they usually do not have the statistical power needed to adequately evaluate uncommon/rare safety outcomes due to Type II (i.e., false negative) error [25]. Given that our review includes rare outcomes, including observational evidence is crucial [26]. Additionally, observational studies can often provide more generalizable evidence due to the strict participant inclusion criteria in most RCTs [27]. Real-world safety evidence that has external validity is important for the assessment of the possible risks of AEDs in pregnant and breastfeeding women.

The diagnosis of neurodevelopmental delay related to *in utero* exposure is made before adolescence, and hence, we will limit inclusion to children up to 12 years of age. AEDs that are approved for chronic use in Canada will be included. Drugs that are only used acutely or those that are not currently approved for use in Canada will be excluded, as the focus of this review is on the Canadian setting [28-32]. However, most of the medications we will examine are available in other countries as well. The relevant 16 medications and their synonyms are listed in Additional file 1, and the excluded drugs are listed in Additional file 2. Studies of all combinations and doses of these medications are eligible for inclusion. Since we are only interested in exposures that occur *in utero* or during breastfeeding, studies examining AEDs administered directly to infants or children will be excluded. All indications for AEDs will be included such as epilepsy, migraine, pain, and psychiatric disorders.

In order to be included, studies must compare an anti-epileptic medication against another included anti-epileptic medication, placebo, a ‘no intervention’ control group, or combinations of two or more anti-epileptic medications. Only studies providing results for our outcomes of interest will be included. Our primary outcome is major congenital malformations (overall and by specific type, such as craniofacial defects and neural tube defects). Secondary outcomes include minor congenital malformations (overall and by specific type, such as epicanthal folds and microstomia), cognition (e.g., global cognitive functioning and specific cognitive domains such as attention), psychomotor development (e.g., autism, dyspraxia), small for gestational age, preterm delivery, neonatal seizures, and fetal loss/miscarriage. No other limitations will be imposed on the eligibility criteria, including published/unpublished material, language of dissemination, duration of follow-up, or year of publication. The draft eligibility criteria can be found in Additional file 3.

### Information sources and literature search

Our main literature search will be executed in the MEDLINE database. The search terms were drafted by an experienced librarian and can be found in Additional file 4. The search was peer reviewed by another librarian using the Peer Review of Electronic Search Strategies checklist [33].

In addition to MEDLINE, we will also search the EMBASE and the Cochrane Central Register of Controlled Trials databases. We will follow the MEDLINE search strategy for these databases, and the search terms will be adjusted accordingly. The electronic database search will be supplemented by searching for unpublished literature [34]. This will be accomplished through exploring conference abstracts, clinical trial registries, and contacting manufacturers of AEDs. We will also scan the reference lists of included studies and previous reviews in the area [23,35,36].

### Study selection process

The eligibility criteria screening form will be pilot-tested by the team and is presented in Additional file 3. We will calculate inter-rater reliability from the pilot-test and screening will only commence after high agreement (e.g., kappa statistic ≥60%) is observed [37]. Subsequently, two reviewers will screen each title/abstract and potentially relevant full-text articles from the literature search results, independently. Conflicts will be resolved through discussion. All screening will occur using our online screening software (synthesi.SR) [38].

### Data items and data collection process

We will abstract data on the PICOS elements [39], including patient characteristics (e.g., age of the mother and infant/child, indication for anti-epileptic treatment, co-morbidities, concomitant medications), intervention details (e.g., type of anti-epileptic treatment, dose, route of administration, duration of treatment, timing [trimester] of treatment during pregnancy), comparator details (e.g., comparator agent, dose, route of administration), outcome results (e.g., major congenital abnormality, minor congenital abnormality, cognitive function, psychomotor development) at the longest duration of follow-up, and study characteristics (e.g., study design, country of conduct, year of conduct, sample size, setting). These characteristics will be abstracted using a data abstraction form created in Excel with an accompanying “cheat sheet” that will guide the reviewers with this process. The data abstraction form and cheat sheet will be pilot-tested and data abstraction will only commence when high agreement (e.g., kappa statistic ≥60%) [37] is observed. Each included study will be abstracted by two team members, independently, who will resolve disagreements through discussion.

### Methodological quality/risk of bias appraisal

We will use various tools to assess the methodological quality/risk of bias of each of the studies that fulfill our eligibility criteria. This will be conducted by two reviewers, independently, and conflicts will be resolved through discussion. First, we will appraise the risk of bias of experimental and quasi-experimental studies using the Cochrane Effective Practice and Organization of Care Risk-of-Bias tool [40]. Second, we will assess the methodological quality of observational studies using the Newcastle-Ottawa Scale [41]. Third, the quality of reporting of harms will be appraised using the McMaster Quality Assessment Scale of Harms (McHarm) tool [42].

### Synthesis of included studies

A narrative summary of study results will be presented along with evidence summary tables. When sufficient data are available, we will conduct random effects meta-analysis to calculate pooled odds ratios for dichotomous data and pooled mean differences for continuous data [43,44]. Direct (pairwise) meta-analysis will be performed with RCTs alone in order to examine whether the data are consistent between direct and indirect evidence. If the large majority of included studies are observational, we will also conduct additional meta-analyses including observational studies alone. Analyses will be stratified by treatment indication (e.g., epilepsy, pain, etc.) to reduce clinical heterogeneity between different study populations whenever possible; for example, epilepsy itself in pregnant women is related to an increased baseline risk of certain neonatal adverse outcomes. Statistical, clinical, and methodological heterogeneity will be examined prior to conducting the meta-analysis. Funnel plots will be drawn for outcomes including at least 10 studies to explore asymmetry that might be explained by clinical, statistical, and methodological heterogeneity. The proportion of statistical heterogeneity will be examined using the I^2^ measure [45] and the magnitude of statistical heterogeneity will be calculated using the restricted maximum likelihood [46]. Meta-regression will be conducted for clinically relevant subgroups or when extensive statistical heterogeneity is observed (e.g., I^2^ ≥ 75%) [47]. This will allow the examination of the impact of important factors on our results, such as maternal age, dose, duration and timing (e.g., trimester) of anti-epileptic treatment, co-morbidities, concomitant medications, risk of bias results, and sample size (due to Type II statistical power errors with rare adverse events). To ensure the meta-regression analysis is intuitive, the number of covariates examined will be less than 10% of the number of studies included in the meta-analysis for the particular outcome.

We anticipate that many of these outcomes will be rare. To deal with studies reporting zero events in one treatment arm, 0.5 will be added to the numerator and 1 will be added to the denominator. We will exclude studies reporting zero events in all treatment arms for a particular outcome [48,49]. We also anticipate that we will encounter missing data in the included studies. We will contact the study authors for this data and if we are unable to receive the data, we will impute missing data (e.g., measures of variance) using established methods [50]. To ensure that our imputations do not bias our results, we will conduct a sensitivity analysis [51]. The meta-analysis and meta-regression will be analyzed in R using the *metafor* command [52].

A random-effects network meta-analysis will be conducted to make inferences regarding the comparative safety of the various AEDs [15], as well as rank their safety using rankograms and the surface under the cumulative ranking curve [53]. We will ensure the following factors are present prior to conducting network meta-analysis: i) transitivity (i.e., comparable distribution of effect modifiers across comparisons), which will be examined using boxplots or percentages to visually inspect potential effect modifiers of treatment effect [54]; ii) consistency between direct and indirect data, which will be examined locally (i.e., in certain paths of the network) using the loop-specific method [55,56] and the node-splitting method [57], and globally (i.e., evaluating the network as a whole), using the design-by-treatment interaction model [58]; and iii) we will quantify the amount of variability attributed to heterogeneity and inconsistency rather than sampling error, by calculating the I^2^[59]. We will estimate the amount of heterogeneity using the restricted maximum likelihood method and assuming common within-network heterogeneity. We will compare the magnitude of heterogeneity between consistency and inconsistency models, as well as between meta-regression and network meta-analysis models to determine how much heterogeneity will be explained by inconsistency or the explanatory variable, respectively. We will first use the design-by-treatment model for the evaluation of inconsistency in a network as a whole and then, if inconsistency is detected, we will employ the loop-specific and node-splitting methods to identify which piece of evidence is responsible for inconsistency. As mentioned above, analyses will be stratified by treatment indication when clinically appropriate. Important heterogeneity and inconsistency will be explored using network meta-regression using the same methods as described above, as necessary.

Prior to conducting the network meta-analysis, we will hold a team meeting to finalize which treatment nodes will be included in the analysis since we are unclear about the indications, dosages, patient populations, and outcomes reported in all of the studies. We will discuss issues, including conducting a class versus independent drug analysis, inclusion of drug routes of administration and dosages, as well as timing of drug administration. These decisions will be examined through a sensitivity analysis in which we will classify treatment nodes using a different classification to see how stable our results are. The network meta-analysis results will be presented as summary treatment effects for each pair of treatments. Network meta-analysis will be conducted in Stata with the *mvmeta* routine [60].

A sequential approach will be used for the network meta-analysis. We will first restrict our analysis to RCTs, which will be the primary analysis of interest. We will then include data from quasi-experimental studies, and finally, data from observational studies. This will provide an understanding of the contribution of each type of study design to our summary estimates, providing us with information on how these agents work above and beyond clinical trials.

## Discussion

Epilepsy is the most common chronic neurological condition, affecting 0.6 to 1% of the population [1,2]. Given that approximately a third of patients receiving AEDs are of reproductive age and almost half of pregnancies are unplanned [61], the fetus may be exposed to these in the first trimester of pregnancy, including during the critical stage of embryogenesis [62].

The comparative safety of these agents is currently unknown and our results will be important for policy-makers, healthcare providers, and women of childbearing age. To ensure our results have wide dissemination and uptake, we will publish our results in open access journals, present our findings at scientific conferences, conduct dissemination meetings with key stakeholders (including policy-makers and healthcare providers), and produce policy briefs for Health Canada, the organization that posed this query.

## Abbreviations

AEDs: Anti-epileptic drugs; RCTs: Randomized clinical trials.

## Competing interests

The authors declare that they have no competing interests.

## Authors’ contributions

ACT conceived and designed the study, helped obtain funding for the study, and helped write the draft protocol. EC registered the protocol with the PROSPERO database and edited the draft protocol. AV helped write the draft protocol. CS edited the draft protocol. BH, BRH, DM, and YF provided input into the design, helped obtain funding for the study, and edited the draft protocol. SES conceived the study, designed the study, obtained the funding, and helped write the draft protocol. All authors read and approved the final protocol.

## Supplementary Material

Additional file 1 List of relevant medications. Click here for file

Additional file 2 Excluded drugs. Click here for file

Additional file 3 Draft eligibility criteria. Click here for file

Additional file 4 MEDLINE literature search. Click here for file
