# Supplementary material for: Comparative safety of anti-epileptic drugs among infants and children exposed in utero or during breastfeeding: protocol for a systematic review and network meta-analysis
Source: Syst Rev. 2014 Jun 25;3:68. doi: 10.1186/2046-4053-3-68 (PMC4086277; doi:10.1186/2046-4053-3-68)
Supplement: Additional file 1 — List of relevant medications. [file 2046-4053-3-68-S1.doc]

**Additional file** 1: List of relevant medications

| **Generic Name** | **Canadian Brand Name** | **Other Names** | |
| --- | --- | --- | --- |
| 1. **Carbamazepine** | Tegretol | Amizepin, Amizepine, Bipotrol, Biston, Carbamazepen, Carbatrol, Carbazepine, Epitol, Equetro, Finlepsin, Karbamazepin, Lexin, Neurotol, Stazepine, Tegretal, Telesmin, Teril, Timonil | |
| 1. **Clobazam** | Frisium | Caastilium, Clobazamum, Noiafren, Odipam, Onfi, Urbadan, Urbanil, Urbanyl | |
| 1. **Clonazepam** | Rivotril | Alti-clonazepam, Antelepsin, Apetryl, Chlonazepam, Cloazepam, Clonapam, Clonex, Clonopin, Iktorivil, Kenoket, Klonopin, Landsen, Lktorivil, Lonazep, Melzap, Paxam, Solfidin | |
| 1. **Ethosuximide** | Zarontin | Aethosuccimidum, Aethosuximide, Asamid, Atysmal, Capitus, Emeside, Epileo, Ethosuccimide, Ethosuccinimide, Ethosuxide, Ethosuximidum, Etomal, Etosuccimide, Etosuximid, Etosuximide, Mesentol, Pemal, Pemalin, Pentinimid, Peptinimid, Petinimid, Piknolepsin, Pyknolepsinum, Ronton, Simatin, Succimal, Succimitin, Suxilep, Suximal, Suxin, Suxinutin, Thetamid, Thilopemal, Zaraondan, Zarodan, Zarondan, Zartalin | |
| 1. **Gabapentin** | Neurontin | Aclonium, Fanatrex, Gabapetin, Gralise, Neuontin, Sefelsa, Serada, Therapentin-90, Vultin | |
| 1. **Lacosamide** | Vimpat | Erlosamide, Harkoseride | |
| 1. **Lamotrigine** | Lamictal | Lamiktal, Lamitor | |
| 1. **Levetiracetam** | Keppra | Etiracetam, Etiracetamum | |
| 1. **Oxcarbazepine** | Trileptal | Oxcarbamazepine, Timox | |
| 1. **Phenobarbital** | Phenobarb | **See list below** | |
| 1. **Phenytoin** | Dilantin | **See list below** | |
| 1. **Primidone** | Primidone | Cyral, Desoxyphenobarbitone, Hexadiona, Hexamidine, Lepimidin, Lepsiral, Liskantin, Majsolin, Medi-Pets, Midone, Milepsin, Misodine, Misolyne, Mizodin, Mizolin, Mylepsin, Mylepsinum, Mysedon, Mysoline, Neurosyn, Prilepsin, Primaclone, Primakton, Primidon, Primidona, Primidonum, Primoline, Prysoline, Sertan | |
| 1. **Rufinamide** | Banzel | Inovelon, Xilep | |
| 1. **Topiramate** | Topamax | Epitomax, Tipiramate, Tipiramato, Topiragen, Trokendi | |
| 1. **Valproic Acid/ Divalproex Sodium** | Depakene/ Epival | Acide valproique, Acido valproico, Acidum valproicum, Convulsofin, Delepsine, Depakane, Depakin, Depakote, Divalproate, Divalproex, DPA, Epilex, Ergenyl, Kyselina 2-propylvalerova, Propylvaleric acid, Sprinkle, Stavzor, Valcote, Valdisoval, Valparin, Valproate, Valproate semisodium, Valproato semisodium, Valproatum semisodium, Vupral | |
| 1. **Vigabatrin** | Sabril | GVG | |
|  | | | |
| 10. **Phenobarbital/ Phenobarb – Other Names:** | | | |
| Adonal | Ensodorm | Liquital | Phob |
| Aephenal | Epanal | Lixophen | Polcominal |
| Agrypnal | Epidorm | Lubergal | Promptonal |
| Amylofene | Epilol | Lubrokal | Quadrinal |
| Antrocol | Episedal | Lumen | Seda-Tablinen |
| Aphenylbarbit | Epsylone | Lumesettes | Sedicat |
| Aphenyletten | Eskabarb | Lumesyn | Sedizorin |
| Austrominal | Etilfen | Luminal | Sedlyn |
| Barbenyl | Euneryl | Lumofridetten | Sedabar |
| Barbidonna | Fenbital | Luphenil | Sedofen |
| Barbiphenyl | Fenemal | Luramin | Sedonal |
| Barbipil | Fenobarbital | Molinal | Sedonettes |
| Barbita | Fenobarbitale | Neurobarb | Sedophen |
| Barbivis | Fenosed | Nirvonal | Sevenal |
| Barbonal | Fenylettae | Noptil | Solfoton |
| Barbophen | Gardenal | Nova-pheno | Solu-Barb |
| Bardorm | Gardepanyl | Nunol | Sombutol |
| Bartol | Glysoletten | Parkotal | Somnolens |
| Bialminal | Haplopan | Pharmetten | Somnoletten |
| Blu-phen | Haplos | Phen-Bar | Somnosan |
| Bronkotabs | Helional | Phenaemal | Somonal |
| Cabronal | Hennoletten | Phenemal | Spasepilin |
| Calmetten | Henotal | Phenemalum | Starifen |
| Calminal | Hydantal | Phenobal | Starilettae |
| Cardenal | Hypnaletten | Phenobarbitalum | Stental |
| Chardonna-2 | Hypnette | Phenobarbitol | Talpheno |
| Chinoin | Hypno-Tablinetten | Phenobarbitone | Tedral |
| Codibarbita | Hypnogen | Phenobarbitonum | Teolaxin |
| Coronaletta | Hypnolone | Phenobarbituric acid |  |
| Cratecil | Hypnoltol | Phenobarbyl |  |
| Dezibarbitur | Hysteps | Phenoluric |  |
| Donnatal | Kinesed | Phenolurio |  |
| Donnazyme | Leonal | Phenomet |  |
| Dormiral | Lepinal | Phenonyl |  |
| Doscalun | Lepinaletten | Phenoturic |  |
| Duneryl | Levsin | Phenyletten |  |
| Ensobarb | Linasen | Phenyral |  |
| 11. **Phenytoin/ Dilantin – Other Names:** | | |  |
| Aleviatin | Epasmir | Neos-hidantoina |  |
| Auranile | Epdantoin | Neosidantoina |  |
| Causoin | Epdantoine | Novantoina |  |
| Comitoina | Epelin | Novophenytoin |  |
| Convul | Epifenyl | OM-Hydantoine |  |
| Danten | Epihydan | Oxylan |  |
| Dantinal | Epilan | Phanantin |  |
| Dantoinal | Epilantin | Phanatine |  |
| Dantoine | Epinat | Phenatine |  |
| Denyl | Epised | Phenatoine |  |
| Di-Hydan | Eptal | Phenhydanin |  |
| Di-Lan | Fenantoin | Phentoin |  |
| Di-Phetine | Fenidantoin | Phentytoin |  |
| Difenilhidantoina | Fenitoina | Phenytoine |  |
| Difenin | Fentoin | Phenytoinum |  |
| Difetoin | Fenylepsin | Ritmenal |  |
| Difhydan | Fenytoin Dak | Saceril |  |
| Dihycon | Fenytoine | Sanepil |  |
| Dihydantoin | Gerot-epilan-D | Silantin |  |
| Dilabid | Hidan | Sinergina |  |
| Dillantin | Hidantal | Sodanthon |  |
| Dintoin | Hidantilo | Sodantoin |  |
| Dintoina | Hidantina | Sodanton |  |
| Diphantoin | Hidantoina | Solantin |  |
| Diphedal | Hidantomin | Sylantoic |  |
| Diphedan | Hydantal | Thilophenyl |  |
| Diphenin | Hydantoinal | Thilophenyl |  |
| Diphenine | Ictalis | Thilophenyl |  |
| Diphentyn | Idantoil | TOIN |  |
| Diphenylan | Idantoin | Tremytoine |  |
| Diphenylhydantoin | Idantoin | Zentronal |  |
| Diphenylhydantoine | Idantoin | Zentropil |  |
| Diphenylhydatanoin | Iphenylhydantoin |  |  |
| Ditoinate | Kessodanten |  |  |
| DPH | Labopal |  |  |
| DPH | Lehydan |  |  |
| Ekko | Lepitoin |  |  |
| Elepsindon | Lepsin |  |  |
| Enkelfel | Mebroin |  |  |
| Epamin | Minetoin |  |  |
