# Supplementary material for: Comparative safety of anti-epileptic drugs among infants and children exposed in utero or during breastfeeding: protocol for a systematic review and network meta-analysis
Source: Syst Rev. 2014 Jun 25;3:68. doi: 10.1186/2046-4053-3-68 (PMC4086277; doi:10.1186/2046-4053-3-68)
Supplement: Additional file 2 — Excluded drugs. [file 2046-4053-3-68-S2.doc]

**Additional file 2: Excluded drugs**

| Acetazolamide |
| --- |
| Diazepam |
| Fosphenytoin |
| Lorazepam |
| Methsuximide |
| Nitrazepam |
| Paraldehyde |
| Piracetam |
| Pregabalin |
| Retigabine |
| Sulthiame |
| Tiagabine |
| Zonisamide |
