# Supplementary material for: Comparative safety of anti-epileptic drugs among infants and children exposed in utero or during breastfeeding: protocol for a systematic review and network meta-analysis
Source: Syst Rev. 2014 Jun 25;3:68. doi: 10.1186/2046-4053-3-68 (PMC4086277; doi:10.1186/2046-4053-3-68)
Supplement: Additional file 3 — Draft eligibility criteria. [file 2046-4053-3-68-S3.doc]

**Additional file 3: Draft Eligibility Criteria**

**Level 1 screening**

1. Does the study include pregnant or breastfeeding women and/or their children/fetuses?

YES____ NO____ UNCLEAR____

1. Were the women treated with anti-epileptic medication? Both monotherapy and combination therapy will be included for any indication.

YES____ NO____ UNCLEAR____

1. Were the women treated with anti-epileptic medication compared to placebo or each other? YES____ NO____ UNCLEAR____
2. Is this a relevant study design (e.g., experimental, quasi-experimental or observational)? YES____ NO____ UNCLEAR____

If you answer NO to any of these questions, the citation will be excluded. All other citations will be included in L2 screening.

**Level 2 screening**

1. Does the study include pregnant or breastfeeding women and/or their children/fetuses that were exposed to anti-epileptic medication *in-utero* or during breastfeeding? YES____ NO____ UNCLEAR____
2. Were the women treated with anti-epileptic medication? Both monotherapy and combination therapy will be included for any indication.

YES____ NO____ UNCLEAR____

1. Were the women treated with anti-epileptic medication compared to placebo or each other? YES____ NO____ UNCLEAR____
2. Does the study report at least one of our safety outcomes of interest (e.g., major congenital malformation, minor congenital malformation, cognitive, psychomotor development, small for gestational age infants, preterm delivery, neonatal seizures, and fetal loss/miscarriage)? YES____ NO____ UNCLEAR____
3. Is this a relevant study design (e.g., experimental, quasi-experimental or observational)? YES____ NO____ UNCLEAR____

If you answer NO to any of these questions, the citation/study will be excluded. All other full-text articles will be included.
