# Supplementary material for: Comparative safety of anti-epileptic drugs among infants and children exposed in utero or during breastfeeding: protocol for a systematic review and network meta-analysis
Source: Syst Rev. 2014 Jun 25;3:68. doi: 10.1186/2046-4053-3-68 (PMC4086277; doi:10.1186/2046-4053-3-68)
Supplement: Additional file 4 — MEDLINE literature search. [file 2046-4053-3-68-S4.doc]

**Additional file 4: MEDLINE literature search**

1 exp Pregnancy/

2 exp Breast Feeding/

3 exp Embryo, Mammalian/

4 exp Fetus/

5 Lactation/

6 Maternal Exposure/

7 Maternal-Fetal Exchange/

8 antenatal$.mp.

9 ante-natal$.mp.

10 antepartum$.mp.

11 ante-partum$.mp.

12 breastfeed$.mp.

13 breast-feed$.mp.

14 breastfed$.mp.

15 breast-fed$.mp.

16 embryo$.mp.

17 fetal.mp.

18 foetal.mp.

19 fetus$.mp.

20 foetus$.mp.

21 gestation$.mp.

22 lactat$.mp.

23 placenta$.mp.

24 perinatal$.mp.

25 perin-natal$.mp.

26 prebirth$.mp.

27 pre-birth$.mp.

28 preterm.mp.

29 pre-term.mp.

30 prepartum$.mp.

31 pre-partum$.mp.

32 prenatal$.mp.

33 pre-natal$.mp.

34 (maternal adj exposure?).mp.

35 (maternal adj2 exchange?).mp.

36 (maternal adj fetal).mp.

37 (maternal adj foetal).mp.

38 (maternal adj fetus$).mp.

39 (maternal adj foetus$).mp.

40 pregnanc$.mp.

41 pregnant.mp.

42 transplacenta$.mp.

43 trans-placenta$.mp.

44 inutero.mp.

45 (in adj utero).mp.

46 intrauterin$.mp.

47 (intra adj uterin$).mp.

48 or/1-47

49 levetiracetamum.mp.

50 levetiracetam.mp,rn.

51 etiracetam.mp.

52 etirazetam.mp.

53 keppra.mp.

54 torleva.mp.

55 UCB-L059.tw.

56 UCB-L060.tw.

57 UCB 6474.tw.

58 Phenytoin/

59 exp Carbamazepine/

60 exp Valproate/

61 exp Phenobarbital/

62 Valproic Acid/

63 Vigabatrin/

64 Clonazepam/

65 Ethosuximide/

66 antelepsin.tw.

67 amizepine.tw.

68 banzel.tw.

69 carbamazepin$.tw.

70 carbazepin.tw.

71 clorepin.tw.

72 chlonazepam.tw.

73 clonazepam$.tw.

74 carbatrol.tw.

75 clobazam.tw.

76 convulsofin.tw.

77 crisomet.tw.

78 22316-47-8.rn.

79 diphenylhydantoin.tw.

80 dilantin.tw.

81 depak?ne.tw.

82 divalproex.tw.

83 depakote.tw.

84 (dipropyl adj acetate).tw.

85 ergenyl.tw.

86 erlosamide.tw.

87 etosuximida.tw.

88 ethosuximide.tw.

89 epamin.tw.

90 epitol$.tw.

91 emeside.tw.

92 epitomax.tw.

93 ethymal.tw.

94 finlepsin.tw.

95 gabapentin$.tw.

96 gardenal.tw.

97 harkoseride.tw.

98 inovelon.tw.

99 klonopin.tw.

100 lamic$.tw.

101 lacosamide.tw.

102 luminal.tw.

103 lamotrig$.tw.

104 lamiktal.tw.

105 labileno.tw.

106 lamitor.tw.

107 (myproic adj acid).tw.

108 neurontin.tw.

109 neurotol.tw.

110 onfi.tw.

111 oxcarbamazepine.tw.

112 oxcarbazepin$.tw.

113 phenytoin.tw.

114 phenobarbital.tw.

115 phenobarbitone.tw.

116 petnidan.tw.

117 phenemal.tw.

118 (propylisopropylacetic adj acid).tw.

119 rivotril.tw.

120 rufinamide$.tw.

121 106308-44-5.rn.

122 sabril$.tw.

123 suxinutin.tw.

124 stavzor.tw.

125 timox.tw.

126 trileptal.tw.

127 tegretol.tw.

128 top?max.tw.

129 topiramat$.tw.

130 97240-79-4.rn.

131 urbanyl.tw.

132 vigabatrin$.tw.

133 60643-86-9.rn.

134 vupral.tw.

135 valproate.tw.

136 (valproic adj acid$).tw.

137 99-66-1.rn.

138 vimpat.tw.

139 xilep.tw.

140 zarontin.tw.

141 etiracetamum.tw.

142 amizepin.tw.

143 bipotrol.tw.

144 biston.tw.

145 carbamazepen.tw.

146 carbazepine.tw.

147 equetro.tw.

148 karbamazepin.tw.

149 lexin.tw.

150 mazepine.tw.

151 stazepine.tw.

152 tegretal.tw.

153 telesmin.tw.

154 teril.tw.

155 timonil.tw.

156 alti-clonazepam.tw.

157 apetryl.tw.

158 cloazepam.tw.

159 clonex.tw.

160 clonopin.tw.

161 clonpam.tw.

162 Iktorivil.tw.

163 kenoket.tw.

164 landsen.tw.

165 lktorivil.tw.

166 lonazep.tw.

167 melzap.tw.

168 paxam.tw.

169 solfidin.tw.

170 caastilium.tw.

171 clobazamum.tw.

172 frisium.tw.

173 noiafren.tw.

174 odipam.tw.

175 urbadan.tw.

176 urbanil.tw.

177 delepsine.tw.

178 divalproate.tw

179 epilex.tw.

180 epival.tw.

181 sprinkle.tw.

182 valcote.tw.

183 valdisoval.tw.

184 valparin.tw.

185 valproato.tw.

186 valproatum.tw.

187 aethosuccimidum.tw.

188 aethosuximide.tw.

189 asamid.tw.

190 atysmal.tw.

191 capitus.tw.

192 epileo.tw.

193 ethosuccimide.tw.

194 ethosuccinimide.tw.

195 ethosuxide.tw.

196 ethosuximidum.tw.

197 etomal.tw.

198 etosuccimide.tw.

199 etosuximid.tw.

200 etosuximide.tw.

201 mesentol.tw.

202 pemal.tw.

203 pemalin.tw.

204 pentinimid.tw.

205 peptinimid.tw.

206 petinimid.tw.

207 piknolepsin.tw.

208 pyknolepsinum.tw.

209 ronton.tw.

210 simatin.tw.

211 succimal.tw.

212 succimitin.tw.

213 suxilep.tw.

214 suximal.tw.

215 suxin.tw.

216 thetamid.tw.

217 thilopemal.tw.

218 zaraondan.tw.

219 zarodan.tw.

220 zarondan.tw.

221 zartalin.tw.

222 aclonium.tw.

223 fanatrex.tw.

224 gabapetin.tw.

225 gralise.tw.

226 neuontin.tw.

227 sefelsa.tw.

228 serada.tw.

229 therapentin-90.tw.

230 vultin.tw.

231 tipiramate.tw.

232 tipiramato.tw.

233 topiragen.tw.

234 trokendi.tw.

235 GVG.tw.

236 (acide adj valproique).tw.

237 (acido adj valproico).tw.

238 (acidum adj valproicum).tw.

239 depakin.tw.

240 DPA.tw.

241 kyselina 2-propylvalerova.tw.

242 (propylvaleric adj acid).tw.

243 Primidone/

244 cyral.tw.

245 desoxyphenobarbitone.tw.

246 hexadiona.tw.

247 hexamidine.tw.

248 lepimidin.tw.

249 lepsiral.tw.

250 liskantin.tw.

251 majsolin.tw.

252 medi-pets.tw.

253 midone.tw.

254 milepsin.tw.

255 misodine.tw.

256 misolyne.tw.

257 mizodin.tw.

258 mizolin.tw.

259 mylepsin.tw.

260 mylepsinum.tw.

261 mysedon.tw.

262 mysoline.tw.

263 neurosyn.tw.

264 prilepsin.tw.

265 primaclone.tw.

266 primakton.tw.

267 primidon$.tw.

268 primoline.tw.

269 prysoline.tw.

270 sertan.tw.

271 aleviatin.tw.

272 auranile.tw.

273 causoin.tw.

274 comitoina.tw.

275 convul.tw.

276 danten.tw.

277 dantinal.tw.

278 dantoinal.tw.

279 dantoine.tw.

280 denyl.tw.

281 di-hydan.tw.

282 di-lan.tw.

283 di-phetine.tw.

284 difenilhidantoina.tw.

285 difenin.tw.

286 difetoin.tw.

287 difhydan.tw.

288 dihycon.tw.

289 dihydantoin.tw.

290 dilabid.tw.

291 dillantin.tw.

292 dintoin.tw.

293 dintoina.tw.

294 diphantoin.tw.

295 diphedal.tw.

296 diphedan.tw.

297 diphenin.tw.

298 diphenine.tw.

299 diphentyn.tw.

300 diphenylan.tw.

301 diphenylhydantoine.tw.

302 diphenylhydatanoin.tw.

303 ditoinate.tw.

304 DPH.tw.

305 ekko.tw.

306 elepsindon.tw.

307 enkelfel.tw.

308 epasmir.tw.

309 epdantoin.tw.

310 epdantoine.tw.

311 epelin.tw.

312 epifenyl.tw.

313 epihydan.tw.

314 epilan.tw.

315 epilantin.tw.

316 epinat.tw.

317 epised.tw.

318 eptal.tw.

319 fenantoin.tw.

320 fenidantoin.tw.

321 fenitoina.tw.

322 fentoin.tw.

323 fenylepsin.tw.

324 (fenytoin adj dak).tw.

325 fenytoine.tw.

326 gerot-epilan-D.tw.

327 hidan.tw.

328 hidantal.tw.

329 hidantilo.tw.

330 hidantina.tw.

331 hidantoina.tw.

332 hidantomin.tw.

333 hydantal.tw.

334 hydantoinal.tw.

335 Ictalis.tw.

336 Idantoil.tw.

337 Idantoin.tw.

338 Iphenylhydantoin.tw.

339 kessodanten.tw.

340 labopal.tw.

341 lehydan.tw.

342 lepitoin.tw.

343 lepsin.tw.

344 mebroin.tw.

345 minetoin.tw.

346 neos-hidantoina.tw.

347 neosidantoina.tw.

348 novantoina.tw.

349 novophenytoin.tw.

350 OM-hydantoine.tw.

351 oxylan.tw.

352 phanantin.tw.

353 phanatine.tw.

354 phenatine.tw.

355 phenatoine.tw.

356 phenhydanin.tw.

357 phentoin.tw.

358 phentytoin.tw.

359 phenytoine.tw.

360 phenytoinum.tw.

361 ritmenal.tw.

362 saceril.tw.

363 sanepil.tw.

364 silantin.tw.

365 sinergina.tw.

366 sodanthon.tw.

367 sodantoin.tw.

368 sodanton.tw.

369 solantin.tw.

370 sylantoic.tw.

371 thilophenyl.tw.

372 TOIN.tw.

373 tremytoin.tw.

374 zentronal.tw.

375 zentropil.tw.

376 adonal.tw.

377 aephenal.tw.

378 agrypnal.tw.

379 amylofene.tw.

380 aphenylbarbit.tw.

381 aphenyletten.tw.

382 austrominal.tw.

383 barbenyl.tw.

384 barbiphenyl.tw.

385 barbipil.tw.

386 barbita.tw.

387 barbivis.tw.

388 barbonal.tw.

389 barbophen.tw.

390 bardorm.tw.

391 bartol.tw.

392 bialminal.tw.

393 blu-phen.tw.

394 cabronal.tw.

395 calmetten.tw.

396 calminal.tw.

397 cardenal.tw.

398 chinoin.tw.

399 codibarbita.tw.

400 coronaletta.tw.

401 cratecil.tw.

402 dezibarbitur.tw.

403 dormiral.tw.

404 doscalun.tw.

405 duneryl.tw.

406 ensobarb.tw.

407 ensodorm.tw.

408 epanal.tw.

409 epidorm.tw.

410 epilol.tw.

411 episedal.tw.

412 epsylone.tw.

413 eskabarb.tw.

414 etilfen.tw.

415 euneryl.tw.

416 fenbital.tw.

417 fenemal.tw.

418 fenobarbital.tw.

419 fenobarbitale.tw.

420 fenosed.tw.

421 fenylettae.tw.

422 gardepanyl.tw.

423 glysoletten.tw.

424 haplopan.tw.

425 haplos.tw.

426 helional.tw.

427 hennoletten.tw.

428 henotal.tw.

429 hypnaletten.tw.

430 hypnette.tw.

431 hypno-tablinetten.tw.

432 hypnogen.tw.

433 hypnolone.tw.

434 hypnoltol.tw.

435 hysteps.tw.

436 leonal.tw.

437 lepinal.tw.

438 lepinaletten.tw.

439 linasen.tw.

440 liquital.tw.

441 lixophen.tw.

442 lubergal.tw.

443 lubrokal.tw.

444 lumen.tw.

445 lumesettes.tw.

446 lumesyn.tw.

447 lumofridetten.tw.

448 luphenil.tw.

449 luramin.tw.

450 molinal.tw.

451 neurobarb.tw.

452 nirvonal.tw.

453 noptil.tw.

454 nova-pheno.tw.

455 nunol.tw.

456 parkotal.tw.

457 pharmetten.tw.

458 phen-bar.tw.

459 phenaemal.tw.

460 phenemalum.tw.

461 phenobal.tw.

462 phenobarb.tw.

463 phenobarbitalum.tw.

464 phenobarbitol.tw.

465 phenobarbitonum.tw.

466 (phenobarbituric adj acid).tw.

467 phenobarbyl.tw.

468 phenoluric.tw.

469 phenolurio.tw.

470 phenomet.tw.

471 phenonyl.tw.

472 phenoturic.tw.

473 phenyletten.tw.

474 phenyral.tw.

475 phob.tw.

476 polcominal.tw.

477 promptonal.tw.

478 seda-tablinen.tw.

479 sedabar.tw.

480 sedicat.tw.

481 sedizorin.tw.

482 sedlyn.tw.

483 sedofen.tw.

484 sedonal.tw.

485 sedonettes.tw.

486 sedophen.tw.

487 sevenal.tw.

488 solfoton.tw.

489 solu-barb.tw.

490 sombutol.tw.

491 somnolens.tw.

492 somnoletten.tw.

493 somnosan.tw.

494 somonal.tw.

495 spasepilin.tw.

496 starifen.tw.

497 starilettae.tw.

498 stental.tw.

499 talpheno.tw.

500 teolaxin.tw.

501 antrocol.tw.

502 barbidonna.tw.

503 bronkotabs.tw.

504 chardonna-2.tw.

505 donnatal.tw.

506 donnazyme.tw.

507 hydantal.tw.

508 kinesed.tw.

509 levsin.tw.

510 quadrinal.tw.

511 or/49-510

512 randomized controlled trial.pt.

513 randomized.mp.

514 placebo.mp.

515 or/512-514

516 Controlled Clinical Trial/

517 Observational Study/

518 (descriptive adj3 stud$).tw.

519 (descriptive adj3 design).tw.

520 (descriptive adj3 analys?s).tw.

521 nonrandom$.tw.

522 non-random$.tw.

523 non-experiment$.tw.

524 nonexperiment$.tw.

525 (natural adj experiment?).tw.

526 (observational$ adj3 stud$).tw.

527 (observational$ adj3 design).tw.

528 (observational$ adj3 analys?s).tw.

529 quasirandom$.tw.

530 quasi-random$.tw.

531 quasiexperimental.tw.

532 quasi-experimental.tw.

533 exp Cohort Studies/

534 Registries/

535 Epidemiologic Methods/

536 limit 535 to yr=1971-1988

537 cohort$.tw.

538 (follow-up adj stud$).tw.

539 (followup adj stud$).tw.

540 (follow-up adj design).tw.

541 (followup adj design).tw.

542 (follow-up adj analys?s).tw.

543 (followup adj analys?s).tw.

544 (follow-up and base-line).tw.

545 (followup and baseline).tw.

546 longitudinal.tw.

547 ("long term" adj stud$).tw.

548 (longterm adj stud$).tw.

549 ("long term" adj design).tw.

550 (longterm adj design).tw.

551 ("long term" adj analys?s).tw.

552 (longterm adj analys?s).tw.

553 (population adj stud$).tw.

554 (population adj analys?s).tw.

555 prospective.tw.

556 retrospective.tw.

557 registry.tw.

558 registries.tw.

559 Cross-Sectional Studies/

560 (cross adj sectional).tw.

561 (incidence adj stud$).tw.

562 (prevalence adj stud$).tw.

563 (transversal adj stud$).tw.

564 exp Case-Control Studies/

565 Control Groups/

566 Matched-Pair Analysis/

567 (case$ adj3 control$).tw.

568 (case adj3 comparison$).tw.

569 (case$ and series).tw.

570 case-referent.tw.

571 (control$ adj3 stud$).tw.

572 (control adj group$).tw.

573 before-after.tw.

574 "before and after".tw.

575 (before adj after).tw.

576 (time adj series).tw.

577 Evaluation Studies/

578 Comparative Study/

579 Intervention Studies/

580 Multicenter Study/

581 Pilot Projects/

582 Program Evaluation/

583 Validation Studies/

584 (comparative adj stud$).tw.

585 (comparison adj stud$).tw.

586 (evaluation adj stud$).tw.

587 effectiveness.tw.

588 intervention.tw.

589 (multicenter adj stud$).tw.

590 (multi-center adj stud$).tw.

591 (multicenter adj stud$).tw.

592 (multi-center adj stud$).tw.

593 (multidimensional adj stud$).tw.

594 (multi-dimensional adj stud$).tw.

595 (pre- adj5 post-).tw.

596 (pretest adj5 posttest).tw.

597 (program$ adj6 evaluat$).tw.

598 or/516-534,536-597

599 515 or 598

600 48 and 511 and 599

601 exp Animals/ not (exp Animals/ and Humans/)

602 600 not 601
